# Optimal income taxation under monopolistic competition

**DOI:** 10.1007/s00199-022-01463-z

**Published:** 2022-10-05

**Authors:** Alexander Tarasov, Robertas Zubrickas

**Affiliations:** 1 HSE University, 11 Pokrovsky Bulvar, Moscow, Russia 109028; 2 University of Bath, Claverton Down, Bath, BA2 7AY UK; 3 Vilnius University, Sauletekio al. 9, 10222 Vilnius, Lithuania

**Keywords:** Tax policy, Monopolistic competition, Market distortion, Variety effect, Endogenous labor, D43, H21, L13

## Abstract

This paper is concerned with cross-dependencies between endogenous market structure and tax policy. We extend the Mirrlees (Rev Econ Stud 38:175–208, 1971) model of income taxation with a monopolistic competition framework with general additively separable consumer preferences. We show that quantity and variety distortions resulting from the market structure require adjustments to income tax policy, which also needs to be complemented with commodity or firm taxation to achieve the constrained social optimum. We calibrate the model and find that in policy design the failure to account for the market structure results in a welfare loss of 1.77%. Motivated by practical cases, we study a policy regime that is solely based on income taxation. We show that departures from the social optimum can be compensated by lower and less regressive income taxes and a smaller government compared to the regime with income and commodity taxes. We also examine the role of consumer preferences for policy outcomes and show that it is substantially amplified by an endogenous market structure.

**JEL Classification** D43 · H21 · L13

✉ Robertas Zubrickas
r.zubrickas@bath.ac.uk

Alexander Tarasov
atarasov@hse.ru

## 1 Introduction

In the traditional approach to income tax policy design, the policy maker's redistributive and budgetary objectives are considered in isolation from the wider economy, which is taken as exogenously given. This approach has drawn criticism, most notably, from Atkinson (2012) who found the taxation literature to fail to take into account cross-dependencies between market structure and tax policy.[1] Income taxation may not only affect income distribution, but also aggregate supply of labor and demand for products and, hence, market outcomes with implications for welfare. From a different angle, a related question can be raised about the role of income tax policy for mitigating inefficient market outcomes under monopolistic competition (Dixit and Stiglitz 1977; Dhingra and Morrow 2019). In this paper, we study the problem of redistributive tax policy with endogenous market outcomes in a general equilibrium model of monopolistic competition with general additively separable consumer preferences.

The problem addressed in this paper is motivated by the recent changes and trends in market outcomes that are of active research and policy interest. Studies based on US data reveal a rise in price markups and the profit share of income, which is attributed to increased market concentration (Hall 2018; Grullon et al. 2019; De Loecker et al. 2020; Barkai 2020). These developments are closely connected with the findings of declining market entry and the decreased role of dynamic young businesses in the economy (Decker et al. 2014). Higher fixed costs, used to explain declining market entry, point more to higher regulatory and institutional costs, rather than to higher economic costs, suggesting scope for policy intervention (Davis and Haltiwanger 2014; Decker et al. 2014; Gutiérrez and Philippon 2019). The calls for policy intervention are strengthened by the observation that the variety effect of increased market entry on welfare can be as large as the associated price effect from increased competition (Feenstra and Weinstein 2017; Quan and Williams 2018). Lastly, the problem addressed in the present paper closely echoes the dual problem of achieving budgetary objectives and improving market outcomes that most governments faced in the aftermath of the Covid-19 pandemic.

In our paper, we endogenize the product space and prices in the Mirrlees (1971) model of optimal income taxation by extending it with the monopolistic competition framework of Dixit and Stiglitz (1977).[2] We study the public authority's problem of tax policy design to maximize the socially weighted welfare of a population of workers. A worker's utility is determined by her consumption of products and supply of labor. Workers differ in their intrinsic productivity, with less productive workers receiving a larger social weight. In the market equilibrium, the number of products and their prices are endogenously determined by the aggregate amount of labor exerted in the economy, the distribution of disposable income, consumer preferences for varieties, firms' profit-maximizing behavior, and free entry. In our analysis, we distinguish between two policy regimes. In the first regime the government can use direct and indirect taxes

[1] Recently, there has been a growing number of papers that study externalities of income taxation in general and partial equilibrium, which we discuss in Related Literature below.

[2] The monopolistic competition framework introduced by Dixit and Stiglitz (1977) is widely employed in many fields of economics, see Thisse and Ushchev (2018) for a literature review. The endogeneity of the product space also distinguishes our framework from Diamond and Mirrlees (1971).

aimed at both firms and workers, while in the second regime the government uses only income taxes aimed at workers.[3] We solve for the optimal tax policy under different regimes and compare the policy outcomes analytically and quantitatively, contributing to understanding why countries may select different tax policy paths.

With general consumer preferences and endogenous labor supply, the monopolistic market structure implies the interconnected distortions of inefficient market entry and firm output, which, in turn, lead to noncompetitive price markups and inefficient labor supply. We demonstrate that income taxation and one additional tax instrument imposed on the product supply side can achieve the constrained social optimum, which is characterized by public firm ownership. The additional tax instrument can be either indirect commodity tax or direct entry tax, which can be then interpreted as that indirect taxes are not necessary to achieve the constrained social optimum. Commodity tax aims to resolve the distortion related to inefficient firm output, which subsequently also corrects for market entry when supported by necessary adjustments to income taxes. In contrast, entry tax aims at the distortion related to inefficient market entry, which ultimately also corrects for inefficient firm output. Interestingly, as firm underproduction coincides with market over-entry, in the optimum we obtain the subsidization of firms when indirect taxes are used, but the taxation of firms when direct taxes are used. With only income taxes available, tax policy cannot resolve all market inefficiencies and, thus, achieve the constrained social optimum in the general case. For instance, in the event of market over-entry, the optimal income tax rates are increased in proportion to the effect of additional consumption on consumers' strength of preference for varieties but in reverse of tax reductions made to correct for noncompetitive markups.

Using an Expo-Power utility function, which includes constant elasticity of substitution (CES) and constant absolute risk aversion (CARA) utility functions as special cases, we calibrate the model to explore quantitatively the welfare losses from the failure to account for the market structure in policy design and to compare the performance of different tax policy regimes. With nonhomothetic Expo-Power preferences, we allow for the role of income distribution for equilibrium outcomes.[4] Our benchmark is the self-confirming policy equilibrium (SCPE) introduced by Rothschild and Scheuer (2013). This policy equilibrium is the solution to the standard Mirrlees (1971) problem with a market structure taken as given but in conformance with the market equilibrium conditions resultant from the SCPE allocation. We also use the SCPE benchmark to calibrate the parameters of the model.

We find that the failure to account for the market structure results in a welfare loss of 1.77% when all tax instruments are available and in 1.22% when only income taxation is available. The difference in the welfare outcomes between the two regimes can be

[3] This dichotomy of regimes serves the purpose of capturing the dichotomy of regimes observed in practice. In Western European countries, all forms of taxation play a sizable role in tax policy, whereas in the US income taxation is the main instrument of tax policy. The dichotomy of regimes is well illustrated by the difference in Covid-19 economic relief response between the US and Europe. In Europe, VAT exemptions for worst affected sectors constituted a major policy instrument (OECD 2020), whereas its counterpart in the US was direct cash payments to its citizens as provisioned by the 2020 CARES Act.

[4] For the implications of nonhomothetic preferences in monopolistic competition see, for instance, Goryunov et al. (2021) and Hsu et al. (2022).

attributed to market over-entry and higher markups, which cannot be resolved by the means of income taxes only. We can also interpret this difference as a welfare loss due to the *laissez faire* approach of the government with regard to market intervention. At the same time, tax policy based on income taxation can have advantages potentially offsetting its under-performance in welfare comparison. In particular, we find that the optimal tax policy with commodity and income taxation requires a much bigger size of government and higher income tax rates at low incomes needed to pay for producer subsidies.[5]

Furthermore, we do not obtain a "trickle down" effect where taxes are lowered for the rich to improve market outcomes for all. When preference for varieties becomes stronger with more consumption, lowering taxes for the rich can result in market over-entry accompanied by universally higher price markups, thus, hurting overall welfare (see Hsu et al. 2022 for a related outcome in the context of international trade). Further quantitative analysis shows that differences in economic outcomes between the policy regimes match the respective empirical differences between the US and European countries. In particular, we find that the optimal policy based on income taxation results in more market entry, lower income taxes, more labor supply, higher markups, smaller government, and more inequality compared to the optimal policy based on direct and indirect taxation.

In our last quantitative exercise, we explore the role of consumer preferences for tax policy design by re-calibrating the model for CES and CARA utility functions. Consumer preferences can have a direct effect on tax policy design through behavioral response and an indirect effect through the market outcome. The SCPE benchmark with an exogenous market structure captures the direct effect and we observe that relative to the Expo-Power preferences the income tax schedule becomes more progressive for CARA preferences and less progressive for CES preferences. With an endogenous market structure, these differences are substantially amplified and, as a result, so are inaccuracies resulting from misspecified preferences, which further stresses the importance of market structure and consumer preferences on policy outcomes.

The remainder of this paper is organized as follows. After a literature review, we present the model in Sect. 2 and solve it for different policy regimes in Sect. 3. We conduct quantitative analysis in Sect. 4. The proofs of the theoretical results are provided in the "Appendix".

## 1.1 Related literature

There is a growing body of literature that deals with general and partial equilibrium effects of tax policy. A tax reform can affect net wages directly and indirectly if workers' occupational choice, including rent seeking activities, is endogenous as in Stiglitz (1982), Rothschild and Scheuer (2013, 2016), Scheuer (2014), Ales et al. (2015), Lockwood et al. (2017), and Sachs et al. (2020). The externalities of adverse selection and moral hazard in labor markets and their effects on tax policy are studied

[5] This discussion connotes with the policy debate on federal tax reform in the US, where the advantages of commodity taxation over income taxation are deemed insufficient to reform the tax system; see, for instance, the President's Advisory Panel on Federal Tax Reform (2005).

by Golosov and Tsyvinski (2007), Chetty and Saez (2010), and Stantcheva (2014, 2017). Tax policy can create pecuniary externalities with implications for real wages due to its effects on aggregate demand and equilibrium prices (da Costa and Maestri 2019; Kushnir and Zubrickas 2019; Eeckhout et al. 2021; Gürer 2021; Kaplow 2021). The distinctive feature of the present paper is its consideration of the variety or market entry effect under general additively separable consumer preferences that may offset the price effect of pecuniary externalities in tax policy design.

The role of additional varieties for consumer welfare has been the object of study in different strands of literature (Dixit and Stiglitz 1977; Krugman 1979; Romer 1994; Broda and Weinstein 2006; Arkolakis et al. 2008; Bilbiie et al. 2012; Hsu et al. 2022). Welfare gains from product expansion can be decomposed into a direct variety effect and an indirect price effect arising from increased competition. Recent empirical studies find the welfare gain of each effect to be of equal size (Feenstra and Weinstein 2017; Quan and Williams 2018; also see Hausman and Leonard 2002 and Brynjolfsson et al. 2003). This empirical finding suggests a role for the variety effect as important as that for the price effect in policy design. Besides the present paper, the variety effect is incorporated in policy design by Bilbiie et al. (2012, 2019), Bilbiie et al. (2014), Lewis and Winkler (2015), Colciago (2016), Etro (2018). Similarly to our paper but in the representative agent setting, Bilbiie et al. (2019) also demonstrate that the variety effect necessitates policy interventions through direct taxes aimed at firms and workers. Our paper differs from these papers in its study of optimal income tax policy in the Mirrleesian setting with heterogeneous population, imperfect information, and endogenous labor supply.[6]

Our analysis also complements the commodity taxation literature. In the absence of firm profits, Atkinson and Stiglitz (1976) show that commodity taxation is unnecessary when optimal income taxes are employed under the assumption of weak separability of utility between labor and consumption goods (also see Mirrlees 1976). Naito (1999) qualifies this result by showing its dependence on the assumption of constant marginal costs of production. For an encompassing treatment of income and commodity taxation, see Scheuer and Werning (2016). We further qualify the result of Atkinson and Stiglitz (1976) by showing that commodity taxes are zero in the event of efficient market entry as with CES preferences or if market entry taxation is available. Otherwise, commodity taxes are imposed to correct for inefficient market entry even when firm profits are zero and marginal costs of production are constant. The latter finding can be also related to the production efficiency theorem of Diamond and Mirrlees (1971), when applied to the extensive margin of the product space. Finally, Myles (1987, 1989) demonstrate a role for corrective commodity taxation against noncompetitive markups, whereas our findings suggest that commodity taxation can be better suited for correcting inefficient entry whereas income taxes for correcting noncompetitive markups.

6 Bilbiie et al. (2019) also consider an extension with endogenous labor supply, though restricted to the case of CES preferences.

## 2 Model

We construct a model of one-sector monopolistic competition with homogeneous firms and heterogeneous consumers/workers who endogenously decide how many units of labor to supply. There is a unit continuum of workers indexed by productivity type $n$ and distributed according to cumulative distribution $F(n)$ that has density function $f(n) > 0$ with support $[\underline{n}, \overline{n}]$. Worker $n$'s earnings are given by $n\ell(n)$, where $\ell(n)$ is the amount of labor supplied by the worker. Disposable income is given by $y(n) = n\ell(n) - T(n\ell(n))$, where $T(n\ell(n))$ is a labor income tax function. The labor cost is captured by an increasing and convex function $c(\ell)$. There is a continuum of size $N$ of differentiated varieties produced by homogeneous firms, with the consumer price of variety $i$ denoted by $p_i$.

Worker $n$ chooses consumption $q_i(n)$ of each variety $i$ and labor $\ell(n)$ that maximize

$$U(n) = \max \int_0^N u(q_i(n))\, di - c(\ell(n)),$$

where $u$ is a twice differentiable increasing and concave function with $u(0) = 0$, subject to the budget constraint

$$\int_0^N p_i q_i(n) di = y(n). \tag{1}$$

Denoting the Lagrange multiplier by $\kappa(n)$, we find the individual demand $q_i(n)$ from the first-order condition $u'(q_i(n)) = \kappa(n)p_i$ or $q_i(n) = (u')^{-1}(\kappa(n)p_i)$. The aggregate demand for variety $i$ is equal to

$$Q_i \equiv \int_{\underline{n}}^{\overline{n}} q_i(n)dF(n) = \int_{\underline{n}}^{\overline{n}} (u')^{-1}(\kappa(n)p_i)dF(n).$$

Taking into account that the Lagrange multiplier $\kappa(n)$ represents the marginal utility of disposable income, the optimal labor supply is determined by $\kappa(n)n(1-T'(n\ell(n))) = c'(\ell(n))$. In the symmetric case with $p_i = p$ and $q_i(n) = q(n) = y(n)/(Np)$, the optimal labor supply and consumption satisfy

$$\frac{u'(q(n))}{p} n(1 - T'(n\ell(n))) = c'(\ell(n)). \tag{2}$$

In the analysis below, we use the measure of the strength of preferences for varieties. Specifically, we follow Zhelobodko et al. (2012) to define generalized *relative love for variety* as

$$\eta \equiv -\frac{Q}{\int \frac{u'(q(n))}{u''(q(n))} dF(n)} = \frac{1}{\int \left[-\frac{q(n)u''(q(n))}{u'(q(n))}\right]^{-1} \frac{q(n)}{Q} dF(n)},$$

which is the weighted harmonic average of individual relative love for variety.[7] As argued by Zhelobodko et al. (2012) for the case of homogeneous population, when preferences feature an increasing (decreasing) relative love for variety in consumption $q$, consumers perceive varieties as less (more) differentiated when they consume more. This property follows from that the relative love for variety is inversely related to the elasticity of substitution between any given pair of varieties.

Each variety $i$ is produced by a single firm with the marginal and fixed cost of production equal to $k$ and $K$, respectively. Letting $s$ denote a unit tax/subsidy and $S$ a lump sum entry tax/subsidy imposed on firm $i$, the firm's maximization problem can be expressed as

$$\max_{p_i} \Pi(p_i, Q_i) = (p_i - s - k)Q_i - S - K.$$

The first-order condition of profit maximization is given by

$$\eta = \frac{p - s - k}{p} \equiv m, \tag{3}$$

which requires the price markup $m$ be equal to consumers' relative love for variety $\eta$. Lastly, we assume free entry into the market, which implies non-negative profits or

$$(p - s - k)Q \geq K + S. \tag{4}$$

In sum, given the tax policy $(T, s, S)$, the market outcome of the model is characterized by Eqs. (1) and (2) for consumption and labor supply, (3) for price, and (4) for the number of varieties.

## 3 Government

We define social welfare $W$ as a weighted sum of workers' utilities

$$W = \int \psi(n)U(n)dF(n),$$

where $\psi(n) > 0$ is a weight attached to a worker with productivity $n$ with $\int \psi(n)dF(n) = 1$. The objective of the public authority is to design a tax policy that maximizes the social welfare subject to the market conditions (3), (9), and the tax revenue at least as large as the exogenous public expenditure of $G$. In addition, we impose that workers' productivity is their private information and, thus, cannot be conditioned upon. We will distinguish two main cases. In Sect. 3.1, we consider the case when the public authority has all three tax instruments $(T, s, S)$ at its disposal, whereas in Sect. 3.2, we consider the case when only income taxation $T$ is available for

7 Henceforth, when integrating over workers' ability space $[\underline{n}, \overline{n}]$, we will omit the integral limits, $\underline{n}$ and $\overline{n}$, to ease exposition.

the public authority. For policy design, we use the benchmark of constrained social optimum where firm ownership is public but workers' productivity is their private information (the exact definition of constrained social optimum is provided in the "Appendix").

To understand the role of monopolistic market structure for tax policy, it is useful to discuss the market distortions that arise in the model. When labor supply is inelastic, there are two distortions associated with the inefficient number of products and output per firm in the market equilibrium (Dixit and Stiglitz 1977). For instance, if we consider the model with a representative consumer, then, depending on the behavior of $u'(q)q/u(q)$, it can be shown that there are too many/few products in the market and too low/high output per firm (see Dhingra and Morrow 2019). In the case of CES preferences, $u'(q)q/u(q)$ is constant, implying that the market equilibrium is socially optimal. The elastic individual supply of labor adds one more distortion into the model related to inefficient labor supply, which interacts with the market distortions.

To illustrate this interaction, we contrast the case of no government intervention ($(T, s, S) = \mathbf{0}$) with the first best. In the social optimum with symmetric information and public firm ownership, the optimal allocation of consumption and labor supply can be shown to have

$$\frac{u'(q(n))n}{k} = c'(\ell(n)),$$

whereas without government intervention it has from (2)

$$\frac{u'(q(n))n}{p} = c'(\ell(n)).$$

As price $p$ is larger than marginal cost $k$, for given consumption $q(n)$ we have the undersupply of labor in the decentralized market, which implies less market entry and, thus, fewer varieties. As a redistributive tax policy can affect labor supply, the optimal tax policy needs to account for cross-dependencies between labor supply and market structure and resultant inefficiencies.

## 3.1 Optimal tax policy

In this subsection, we let the public authority have at her disposal the full set of instruments: income tax schedule $T(n\ell(n))$, commodity unit tax $s$ (or subsidy when $s < 0$), and entry tax $S$ (or subsidy when $S < 0$). As the public authority observes only workers' labor income, by the Revelation Principle the optimal income tax policy must satisfy the incentive compatibility constraint for each productivity type. In our framework, this condition can be written as follows

$$U(n) = Nu\left(\tfrac{n\ell(n)-T(n\ell(n))}{Np}\right) - c(\ell(n)) \geq Nu\left(\tfrac{n'\ell(n')-T(n'\ell(n'))}{Np}\right) - c\left(\tfrac{n'\ell(n')}{n}\right)$$

for any $n$ and $n'$. In words, a worker with productivity $n$ does not seek the labor income of a worker with productivity $n'$ or, put differently, workers reveal their types truthfully. This in turn implies that the tax function needs to satisfy

$$\frac{1-T'(n\ell(n))}{p} n u'\left(\tfrac{n\ell(n)-T(n\ell(n))}{Np}\right) = c'(\ell(n)), \tag{5}$$

which is equivalent to the labor supply condition in (2). Using the envelope theorem, we have that

$$U'(n) = \frac{\ell(n)}{p} u'\left(\tfrac{n\ell(n)-T(n\ell(n))}{Np}\right)(1-T'(n\ell(n)))$$

or, using (5), that

$$U'(n) = \frac{\ell(n)}{n} c'(\ell(n)).$$

In subsequent analysis, we will use the latter expression for the incentive compatibility constraint. Lastly, in addition to the second-order condition for the individual optimal allocation in (2), we require labor income $n\ell(n)$ be non-decreasing in productivity $n$ to ensure the second-order condition for the optimality of truth-telling (Mirrlees 1976). As in most applications of the Revelation Principle, we omit this monotonicity constraint from the formulation of the public authority's optimization problem, but we check this constraint in our numerical simulations.

As it is standard in the taxation literature, rather than solving for the optimal income tax policy $T(n\ell(n))$, we will solve for the optimal consumption and labor supply allocation, from which the optimal taxes can then be derived. For analytical convenience, we present the public authority's problem maximized over utility $U(n)$, labor $\ell(n)$, number of varieties $N$, price $p$ with the consumption of each variety $q(n)$ then found from

$$q(n) = u^{-1}\left(\tfrac{U(n)+c(\ell(n))}{N}\right) \equiv r\left(U(n), \ell(n), N\right).$$

The public authority solves the following constrained optimization problem:

$$\max_{U(n),\ell(n),p,N,s,S} \int \psi(n)U(n)dF(n) \tag{6}$$

subject to

$$U'(n) - \frac{\ell(n)}{n} c'(\ell(n)) = 0 \tag{7}$$

$$\int [n\ell(n) - N(p-s)\, r(U(n), \ell(n), N)]dF(n) + NS \geq G \tag{8}$$

$$(p-k-s)\int r(U(n), \ell(n), N)dF(n) - K - S \geq 0 \tag{9}$$

$$\eta - \frac{p-k-s}{p} = 0, \tag{10}$$

where (7)–(10) are the incentive compatibility, resource, free entry, and market price constraints, respectively.

In characterizing the optimal tax policy, it is useful to introduce, following Dhingra and Morrow (2019), a generalized *social markup* defined as

$$\delta \equiv 1 - \frac{\int q(n)dF(n)}{\int \frac{u(q(n))}{u'(q(n))}dF(n)}. \tag{11}$$

It captures workers' net utility from consumption of an additional variety. Keeping everything else constant, an increase in the number of varieties $N$ affects each worker's utility from consumption in two ways. First, there is a new variety effect that increases each worker's welfare by $u(q)$. Second, an increase in $N$ implies a smaller amount $q$ of each variety consumed, which reduces welfare by $q(n)u'(q(n))$. The concavity of $u(q)$ together with $u(0) = 0$ imply that we have $u(q(n))/u'(q(n)) > q(n)$ or $\delta > 0$ or, in words, a dominant first effect and, accordingly, utility increasing in the number of varieties given the levels of workers' income. From a different angle, we note that the variety effect is related to market entry distortions, whereas the price effect to firm output distortions.

The next proposition presents our main theoretical result about optimal tax policy $(T, s, S)$. Following Saez (2001), we present the optimal marginal income taxes, $t(n\ell(n)) = T'(n\ell(n))$, in the elasticity form. For this purpose, we denote uncompensated and compensated labor supply elasticity by $\zeta^u$ and $\zeta^c$, respectively. In the proof of the proposition, we provide the exact formulas for labor supply elasticities and their derivation.

**Proposition 1** *(i) The optimal tax policy consisting of income tax schedule $T(n\ell(n))$ and commodity unit tax $s$ or entry tax $S$ implements the constrained social optimum. (ii) If $s = 0$ and $m \lesseqqgtr \delta$, then $S \lesseqqgtr 0$; if $S = 0$ and $\delta \lesseqqgtr \eta$, then $s \lesseqqgtr 0$. (iii) The optimal marginal income tax $t(n\ell(n)) = T'(n\ell(n))$ for income level $n\ell(n)$ is found from*

$$\frac{t(n\ell(n))}{1 - t(n\ell(n))} = \frac{1 + \zeta^u}{\zeta^c} \frac{\kappa(n)}{nf(n)} \int_n^{\overline{n}} \frac{1 - \kappa(n')\psi(n')/\lambda - \frac{p-k}{p}}{\kappa(n')} dF(n') - \frac{p-k}{p}, \tag{12}$$

*where the marginal utility of disposable income $\kappa(n) = u'(q(n))/p$ and $\lambda$ is the Lagrange multiplier of the resource constraint (8).*

***Proof*** In the "Appendix". □

In part (i) of the proposition, we show that the public authority can achieve the constrained social optimum with income taxation complemented with just one tax instrument for the product supply side: indirect commodity tax or direct entry tax. This finding can also be interpreted that indirect taxation is not necessary for correcting market distortions if entry taxation is feasible. In part (ii), we provide conditions for the taxation or subsidization of the product supply side. For instance, if the market outcome has the price markup larger than the generalized social markup, $m > \delta$, or, put differently, if an additional variety is socially inefficient, then the public authority imposes an entry tax $S > 0$ to reduce market entry. From a different perspective, if the market outcome has the relative love for variety larger than the generalized social markup, $\eta > \delta$, i.e., if the utility from increased consumption of given varieties exceeds the utility from introduction of a new variety, then the public authority offers a commodity subsidy $s < 0$ to increase individual firm output. Interestingly, since market over-entry can coincide with underproduction, firms' taxation or subsidization depends on the instrument chosen to achieve the social optimum: with direct taxes, it would be taxation, but with indirect taxes – subsidization. As we show later with numerical simulations, these two policy types also have very different implications for the optimal income taxation.

Furthermore, as we demonstrate in the proof, in the optimum the benefit from encouraging further entry, given by the multiplier $\alpha$ of the free entry condition (9), needs to be equal to the resultant welfare costs, given by the shadow price of public funds $\lambda$ multiplied by the number of firms $N$. The optimality condition $\alpha = \lambda N$ will be relevant for our subsequent analysis and, in general, is reminiscent of the production efficiency principle of Diamond and Mirrlees (1971) when applied to the number of varieties, i.e., production also needs to be efficient on the extensive margin of product supply. We note that in the case of CES preferences, the social markup is equal to the price markup, implying zero entry or commodity taxes. The latter in turn means that income taxation is sufficient for achieving the socially optimal allocation as stated in the next proposition.

The production inefficiencies related to the presence of noncompetitive markups and endogenous labor supply are corrected by the means of income taxation. Absent price markups ($p = k$), the marginal income tax formula in (12) reduces to the classical Mirrlees (1971) formula obtained under an exogenous market structure. It is determined by (i) workers' behavioral response to taxation captured by labor supply elasticities ($\zeta^u$, $\zeta^c$) and income effects ($\kappa$) integrated over the supramarginal types in $[n, \overline{n}]$ that are affected by marginal tax $t(n\ell(n))$, (ii) the shape of productivity distribution ($F$), and (iii) social concerns ($\psi$). With positive markups ($p > k$), the income tax rates are reduced to encourage more labor supply and, in turn, more production by firms. We note that the adjustment to income tax rates is made with regard to the markup based on consumer price $p$ rather than on producer price $p - s$ and also applies to the incentive term, i.e., the first term of the tax formula in (12).

### 3.2 Optimal income tax policy

In this subsection, we study the case when the public authority resorts only to income taxation with commodity and entry taxes correspondingly set to zero. Under the *laissez faire* approach to market intervention, the public authority's maximization problem reads as

$$\max_{U(n),\ell(n),p,N} \int \psi(n)U(n)dF(n) \tag{13}$$

subject to

$$U'(n) - \frac{\ell(n)}{n} c'(\ell(n)) = 0 \tag{14}$$

$$\int [n\ell(n) - Np\,r(U(n), \ell(n), N)]dF(n) \geq G \tag{15}$$

$$(p-k) \int r(U(n), \ell(n), N) dF(n) - K \geq 0 \tag{16}$$

$$\eta - \frac{p-k}{p} = 0. \tag{17}$$

Let $\lambda$, $\alpha$, and $\beta$ denote the Lagrange multipliers of the resource, free entry, and market price constraints, (15)–(17), respectively. We can formulate the following proposition about the optimal tax policy solely based on income taxation $T(n\ell(n))$.

**Proposition 2** *(i) Under the regime based on income taxation, the optimal tax policy implements the constrained social optimum only for constant elasticity of substitution (CES) preferences. (ii) The optimal marginal income tax $t(n\ell(n))$ for income level $n\ell(n)$ is found from*

$$\frac{t(n\ell(n))}{1 - t(n\ell(n))} = \frac{1+\zeta^u}{\zeta^c} \frac{\kappa(n)}{nf(n)} \int_n^{\bar{n}} \frac{1 - \kappa(n')\psi(n')/\lambda - E(n')}{\kappa(n')} dF(n') - E(n), \tag{18}$$

*where*

$$E(n) = \frac{\alpha}{\lambda N} \frac{p-k}{p} + \frac{\beta}{\lambda p N} \frac{\partial \eta}{\partial q(n)} \tag{19}$$

*and $sign(\beta) = sign(\alpha - \lambda N)$.*

***Proof*** The proof of the first statement follows from the second statement in Proposition 1, as only for CES preferences we have that $\delta = \eta$ or that product supply side taxation is not needed for achieving the constrained social optimum. The proof of the second statement is given in the "Appendix". □

The optimal marginal income tax formula in (18) takes a form similar to that in Proposition 1. The difference is that in Proposition 2 the additional tax term $E(n)$ aims to account for all market inefficiencies arising from inefficient entry, output per firm, and labor supply. But as part (i) of the proposition shows, it is impossible to achieve in the general case. The sign of $E(n)$ depends on the signs of the Lagrange multipliers ($\lambda$, $\alpha$, and $\beta$) and the properties of relative love for varieties captured by the size and sign of $\partial\eta/\partial q(n)$. By the Kuhn-Tucker conditions, we have multipliers $\lambda \geq 0$ and $\alpha \geq 0$, whereas the sign of $\beta$ has to be analytically determined (because it is associated with the equality constraint). In words, we note that $\lambda$ represents the marginal utility of the public authority's spending, $G$, given by $-\lambda$. Since higher spending reduces workers' utility, multiplier $\lambda$ is positive. In a similar way, $-\alpha$ stands for the marginal utility of the fixed entry costs $K$, which is negative, implying positive $\alpha$.

As in Proposition 1, we find that income tax rates are lowered to correct for the inefficient production related to positive markups, $p-k$, which is captured by the first term of $E(n)$ in (19). The role of the second term of $E(n)$ is to account for the variety effect related to free market entry. In the event of market over-entry (with $\alpha < \lambda N$ and, therefore, $\beta < 0$), which is typical for monopolistic competition models with pro-competitive entry effects, and absent other instruments, the public authority stimulates market exit by manipulating price markups through the demand side. Recalling that in the equilibrium the price markup equals relative love for varieties $\eta$, we obtain a further decrease in tax rates if the partial derivative of $\eta$ with respect to $q(n)$ is negative, and an increase in tax rates if the derivative is positive. This mechanism works in reverse for market under-entry ($\alpha > \lambda N$ and, thus, $\beta > 0$). We also note that unlike the first term the second term of $E(n)$ is variable as

$$\frac{\partial \eta}{\partial q(n)} = -\frac{1+\left(\frac{u'(q(n))}{u''(q(n))}\right)'\eta}{\int \frac{u'(q(n))}{u''(q(n))}dF(n)}.$$

Thus, it can have a variable effect on tax rates across income groups, with the degree of variation determined by $\left(u'(q)/u''(q)\right)'$. For instance, if the latter is positive, then $\partial\eta/\partial q(n)$ is also positive, implying lower (higher) tax rates for positive (negative) $\beta$. In the case of CES or constant absolute risk aversion (CARA) preferences, the derivative $\left(u'(q)/u''(q)\right)'$ is constant, implying constant $E(n)$ across income groups.

## 4 Calibration

In this section, we numerically analyze the effect of the endogenous market structure on income tax rates. As our analysis shows that with only income taxation the public authority cannot generally achieve the constrained social optimum, we also attempt to quantify the welfare loss due to the policy design restriction.[8]

### 4.1 Benchmark

We calibrate the model using the self-confirming policy equilibrium (SCPE) introduced by Rothschild and Scheuer (2013) as a benchmark. The concept of SCPE offers an approach to evaluate welfare losses from failing to take into account the endogeneity of some economic attributes. In our case, the SCPE is the solution to the standard Mirrleesian problem of optimal income taxation with the market structure, captured by number of firms $N$ and market price $p$, falsely taken as exogenously given. At the same time, we impose that the market price and the number of firms need to coincide with the equilibrium conditions for market price and zero profits, which would falsely

[8] In the previous section, we do not address the question whether the solution to the system of the first-order conditions that characterize the optimum is unique. While the issue of uniqueness is of no consequence for the qualitative results discussed before, it may be of a concern for numerical comparative analysis. We addressed this concern by employing different numerical methods and robustness checks (like perturbing initial values), which all confirmed the results we report below.

confirm the optimality of the SCPE tax schedule. Hence, while by design the SCPE income tax schedule and the optimal income tax schedule satisfy the same incentive, resource, and market equilibrium conditions, the two schedules may yet be different with the difference solely arising from the endogeneity of the market structure. Put differently, the SCPE schedule overlooks the impact of tax policy on market structure and, as a result, the welfare implications of this impact.

Technically, we use the equilibrium conditions for market price and zero profits as a part of the system of equations that determines the SCPE but set their associated Lagrange multipliers at 0. Hence, the government solves

$$\max_{U(n),\ell(n)} \int \psi(n)U(n)dF(n)$$

subject to

$$U'(n) - \frac{\ell(n)}{n}c'(\ell(n)) = 0$$

$$\int [n\ell(n) - Npr\,(U(n), \ell(n), N)]\,dF(n) \geq G \tag{20}$$

taking $N$ and $p$ as given. At the same time, the latter variables need to satisfy

$$(p-k)\int r(U(n), \ell(n), N)dF(n) - K = 0,$$

$$\eta - \frac{p-k}{p} = 0.$$

We choose the parameters in such a way that some moments associated with the above equilibrium fit those in the data (see details below). We also note that in the SCPE, as in the standard Mirrleesian framework, the optimal commodity tax is zero. The calibrated parameters are then used in the numerical simulations of optimal taxation with the endogenous market structure under the scenarios when (i) only income taxes are available and (ii) income and commodity taxes are available.

### 4.2 Parameters

We model consumer preferences using the two-parameter Expo-Power utility function

$$u(q) = \frac{(1 - e^{-\gamma q^{1-\rho}})}{\gamma}, \tag{21}$$

where $\gamma \geq 0$ and $1 > \rho \geq 0$ are parameters to calibrate (Saha 1993). Note that this utility specification captures two widely used specifications: the case of $\gamma \to 0$ corresponds to the CES utility function, and the case of $\rho = 0$ to the CARA utility function. We calibrate the values of $\gamma$ and $\rho$ using the conditions for the price elasticity of aggregate demand $\varepsilon$ defined by

$$\varepsilon \equiv -\frac{dQ}{dp}\frac{p}{Q} = -\frac{\int u'(q(n))/u''(q(n))dF(n)}{\int q(n)dF(n)}$$

and for pass-through elasticity $\varepsilon_{pk}$ defined by

$$\varepsilon_{pk} \equiv \frac{d\log p}{d\log k} = 1 + \frac{d\log}{d\log k}\left(\frac{\varepsilon}{\varepsilon - 1}\right).$$

We set the price elasticity at $\varepsilon = 4$ in order to match the time average for markups equal to 1.33 (see De Loecker et al. 2020). The estimates of the pass-through elasticity vary in the range between 0.3 and 0.8 and we set $\varepsilon_{pk} = 0.6$ at the rounded midpoint of this range (see Kichko and Picard 2020), which is also the value estimated by Campa and Goldberg (2005) and Amiti et al. (2019).

Productivity $n$ and its distribution $F$ are proxied by hourly wages and its empirical distribution is taken from Mankiw et al. (2009). We set $\underline{n} = 0$ with its mass at 5% of the population to account for economically inactive people. The labor cost function is given by $c(\ell) = \ell^3/3$, which corresponds to the Frisch labor supply elasticity of 0.5 (Chetty et al. 2013). The marginal cost of production $k$ is set to be equal to the reciprocal of the average hourly wage $1/\int n dF(n)$ similarly to Behrens et al. (2020).

We calibrate the fixed cost of production $K$ to match the estimated welfare effects of introducing a new variety. There are a direct variety effect due to the expansion of variety selection and an indirect price effect due to the price change caused by a higher level of competition. In particular, recall that the utility from individual consumption can be written in the case of homogeneous firms as $Nu\,(y(n)/Np)$. Based on that, we define the variety effect (keeping the disposable income fixed) as

$$\int \frac{\partial}{\partial N}\left[Nu\left(\tfrac{y(n)}{Np}\right)\right]dF(n) = \int \left[u(q(n)) - q(n)u'(q(n))\right]dF(n), \tag{22}$$

while the price effect is given by

$$\int N\frac{\partial u\left(\frac{y(n)}{Np}\right)}{\partial p}\frac{dp}{dN}dF(n) = -\int q(n)u'(q(n))\frac{dp}{dN}\frac{N}{p}dF(n), \tag{23}$$

where $dp/dN$ is the implicit derivative of the profit maximizing price with respect to the number of firms $N$. In our calibration, we draw on Feenstra and Weinstein (2017) and Quan and Williams (2018) who show that the price and variety effects are of a similar size. As a result, we set $K$ such that the value of the variety effect is the same as that of the price effect in the SCPE.

Finally, we assume that the government intervenes out of considerations for redistribution only and, therefore, we set its exogenous expenditures at $G = 0$. We follow Rothschild and Scheuer (2013) in modeling the social welfare weights by $\psi(n) = \iota(1 - F(n))^{\iota-1}$ with $\iota = 1.3$.

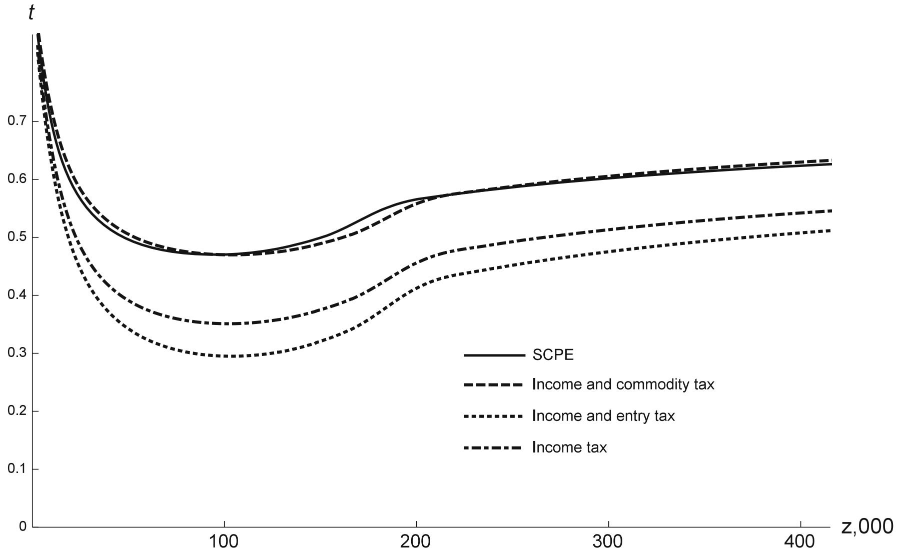

**Fig. 1** Income tax rates and policy regimes. The income tax rate schedules for the policy with income and commodity tax (dashed line) and with income and entry tax (dotted line) are found from Proposition 1 and for the policy with only income tax (dash-dotted line) from Proposition 2. The SCPE tax schedule (solid line) is found from the solution to problem (20)

## 4.3 Results and discussion

### 4.3.1 Self-confirming policy

The calibration of the benchmark SCPE case yields the following parameters values: $\gamma = 0.655$ and $\rho = 0.091$ for the utility function and $K = 0.005$ for the fixed costs of production. The first observation is that calibrated consumer preferences do not exhibit constant elasticity of substitution as parameter $\gamma$ is different from zero. In Fig. 1, for different regimes we plot the optimal marginal income tax rates against gross earnings $z$, where the earnings of the median consumer are normalized to be equal to the empirical median earnings in the US. In the SCPE, the marginal income tax schedule (solid line) takes a shape familiar to those reported in the related literature (Saez 2001; Mankiw et al. 2009). The high tax rates at low incomes capture the phase-out of transfer payments and, in practice, correspond to the rates at which transfers are withdrawn as claimants' earnings increase. The kink at high incomes with the flattening of tax rates is due to the Pareto tail of the productivity distribution.

### 4.3.2 Optimal tax policy

According to Proposition 1, two distinct policy designs can lead to the constrained social optimum. One policy design uses direct (income) and indirect (commodity) taxes, and the other policy design uses only direct (income and entry) taxes. In this subsection, we analyze both designs with a focus on implications for income tax rates.

**Table 1** Numerical outcomes of tax policy regimes

| Tax policy | $W$ | $m$ | $s$ | $S$ | $N$ | $\overline{q}$ | $L$ | $T/L$ | $CV$ |
|---|---|---|---|---|---|---|---|---|---|
| *Expo-power preferences* | | | | | | | | | |
| SCPE | 3651 | 0.33 | 0 | 0 | 20,890 | 0.22 | 295 | 0.57 | 0.42 |
| Income/commodity | 3715 | 0.29 | − 0.012 | 0 | 20,217 | 0.25 | 320 | 0.58 | 0.47 |
| Income/entry | 3715 | 0.38 | 0 | 0.001 | 20,217 | 0.25 | 320 | 0.51 | 0.47 |
| Income | 3695 | 0.34 | 0 | 0 | 23,018 | 0.21 | 322 | 0.48 | 0.49 |
| *CES preferences* | | | | | | | | | |
| SCPE | 5331 | 0.33 | 0 | 0 | 16,871 | 0.31 | 337 | 0.56 | 0.53 |
| Income/commodity | 5455 | 0.33 | 0 | 0 | 19,148 | 0.31 | 383 | 0.43 | 0.68 |
| Income/entry | 5455 | 0.33 | 0 | 0 | 19,148 | 0.31 | 383 | 0.43 | 0.68 |
| Income | 5455 | 0.33 | 0 | 0 | 19,148 | 0.31 | 383 | 0.43 | 0.68 |
| *CARA preferences* | | | | | | | | | |
| SCPE | 2728 | 0.33 | 0 | 0 | 35,059 | 0.12 | 265 | 0.58 | 0.39 |
| Income/commodity | 2775 | 0.28 | − 0.016 | 0 | 32,714 | 0.14 | 282 | 0.63 | 0.42 |
| Income/entry | 2775 | 0.42 | 0 | 0.001 | 32,714 | 0.14 | 282 | 0.55 | 0.42 |
| Income | 2754 | 0.33 | 0 | 0 | 37,856 | 0.12 | 287 | 0.50 | 0.44 |

$W$, Welfare; $m = (p - k - s)/p$, Producer price markup; $s$, Commodity tax/subsidy; $S$, Entry tax/subsidy; $N$, Number of firms; $\overline{q}$, Average consumption; $L$, Total labor earnings; $T/L$, Ratio of total tax revenue to total labor earnings; $CV$, Coefficient of variation of utility (standard deviation divided by average utility)

We observe that the optimal policy regime with income and commodity taxation results in the subsidization of firms by the means of unit subsidy $s = -0.012$ (see the Expo-Power preferences section of Table 1 for numerical policy outcomes). This, in turn, implies from Proposition 1 that without government intervention there is underproduction of individual varieties, which can also be seen from the lower value of average consumption $\overline{q}$ of individual varieties under the SCPE outcome. At the same time, we obtain the marginal income tax schedule (dashed line) that is very similar to the SCPE schedule. We recall from Proposition 1 that production inefficiencies related to noncompetitive markups need to be corrected by lowering marginal tax rates. The markup effect is, however, offset by the firm output distortion that requires additional tax revenue and, thus, higher income taxes to pay for firms' subsidization. Notwithstanding the similarity of the tax schedules, under the optimal tax policy the overall effect of market distortions on tax rates is to the relative disadvantage of consumers with lower incomes, as they face higher tax rates by up to several percentage points. The resultant welfare improvement over the SCPE outcome is about 1.77%, which, as already suggested by changes in tax rates, is not evenly distributed, as we obtain an increase in the coefficient of variation of utility (see column $CV$ in Table 1).

When commodity taxation is replaced with market entry taxation, we obtain a very different tax structure despite that under both designs the constrained social optimum is achieved. In particular, instead of subsidization we obtain the taxation of firms because entry tax $S$ aims to reduce the number of varieties, which will also increase the consumption of individual varieties.[9] Furthermore, entry taxation has also very different implications for the optimal income tax rates. As can be seen from Fig. 1, the marginal income tax rates (the dotted line) are substantially lower than those in the case with commodity taxation. This can be explained by the additional tax revenue that the government collects from entry taxation. At the same time, the policy with entry taxation requires a smaller size of the government, $T/L$, compared to the policy with commodity taxation.

All in all, the possibility of market intervention provides the government with two quantitatively and qualitatively different options to achieve the constrained social optimum. The first one involves a commodity subsidy and relatively high marginal income tax rates, whereas the second one involves an entry tax and lower marginal income tax rates. The latter option also requires a smaller government.

#### 4.3.3 Optimal income tax policy

In this subsection, we study the numerical outcomes of the policy regime based only on income taxation. To recall, our policy assumption is that the government pursues the *laissez faire* approach with regard to product markets. The previous analysis of the two optimal policy regimes reveals some practical disadvantages of market intervention. In particular, the policy with commodity taxation results in higher and less progressive income tax rates and, consequently, in a larger government. The other policy requires a market entry tax, which in the optimum is quite sizable: 20% of the fixed costs.

The purpose of the correction term $-E(n)$ in the income tax formula given in Proposition 2 is to correct for the welfare effects of noncompetitive markups and market inefficiencies. Given market over-entry, which we earlier demonstrated, the two effects work in opposite directions. On the one hand, income taxes need to be increased to reduce market entry and, thus, the number of varieties, but on the other hand they need to be decreased to offset production inefficiencies related to noncompetitive markups. When measured against the SCPE benchmark, we see that the markup effect is dominating as there is a steep reduction in income tax rates. It is noteworthy that the largest reduction of about 10% points occurs at tax rates for low incomes, with the level of reduction gradually diminishing for higher incomes. Formally, as shown in Proposition 2, the size of tax change is proportional to the effect of additional consumption on relative love for varieties $\eta$. As $\partial\eta/\partial q$ is positive and larger at higher incomes or, in other words, richer consumers have an increasingly stronger love for varieties, tax rates are corrected upwards more for higher incomes (see the solid line in Fig. 2 for the plot of $-E(n)$ term under Expo-Power preferences). Thus, we obtain more progressivity in tax rates for reasons other than income redistribution. Put differently, the "trickle-down" effect from less progressive taxes can lead to inefficient entry with more varieties but, inevitably, also with higher price markups and welfare losses (for a related finding also see Hsu et al. 2022).

[9] With only one income class, it is possible to show that in the case of the Expo-Power preferences, the price markup is always higher than the social one, implying over-entry into the market (see Dhingra and Morrow 2019). Our calibration exercise, in turn, shows that this relationship between the markups can hold for the case with many income classes as well.

Comparing welfare outcomes, we find that the income taxation policy under-performs the optimal tax policy by 0.55% relative to the SCPE outcome. This under-performance can be attributed to market over-entry and lower quantities which are impossible to resolve by the means of income taxes only. Yet, from a practical perspective it is not unequivocal which tax regime the government may prefer as market intervention with entry taxation (20% of the fixed costs in the optimum) or production subsidies coupled with regressive income adjustments may appear unpopular in practice. Hence, we can interpret our findings as that the price for the *laissez faire* approach to market intervention is a welfare loss of 0.55%. Furthermore, the income taxation policy comes with progressive marginal tax reductions and, as a result, a much smaller government (see column $T/L$ in Table 1), which may be politically more appealing. Lastly, the observed differences in the quantitative outcomes between the regime with income and commodity taxation and the regime with income taxation match well the corresponding differences across countries where different regimes are practiced. In the US, where the main tax instrument is income taxation, there are higher markups, more entrepreneurs and income per capita, and more inequality, but smaller government compared to Western European countries where both income and commodity taxation are widely used.[10]

### 4.4 Role of preferences

In this subsection, we examine the role of consumer preferences for policy outcomes. Besides Expo-Power preferences, we consider two commonly used types of preferences: CES and CARA. As these preferences are characterized with a one-parameter utility function, we accordingly need one moment condition less and, for this purpose, remove the condition on pass-through elasticity $\varepsilon_{pk} = 0.6$. Furthermore, CES preferences imply the constant ratio of the variety and price effects, respectively defined by (22) and (23), which therefore cannot then be used to calibrate fixed costs of production $K$. Therefore, for CES preferences we take $K = 0.005$ calibrated under Expo-Power preferences.

The left and right panels of Fig. 3 plot the optimal marginal income tax schedules for CES and CARA preferences, respectively, under the regimes of SCPE, income and commodity taxation, and only income taxation. Table 1 reports numerical economic outcomes. As theoretically predicted, with CES preferences the policy outcomes coincide under the regimes with commodity and income taxation and with only income taxation. The correction of the inefficiency related to noncompetitive price markups also ensures the efficiency of market entry, implying the sufficiency of income tax policy. Thus, under both regimes after the phase-out stage of transfer payments we obtain a uniform reduction in income tax rates compared to the SCPE benchmark. From a different perspective, the market correction term $-E(n)$ is constant and negative as shown in Fig. 2 (dotted line).

With CARA preferences, the policy outcomes differ because the income tax instrument alone cannot rectify both market entry and noncompetitive markup inefficiencies.

[10] See Aquilante et al. (2019) for empirical evidence on markups, GEM (2020) on entrepreneurship, and, e.g., the OECD on country statistics.

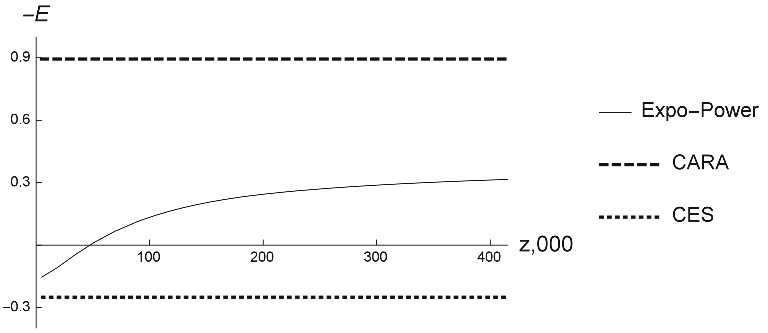

**Fig. 2** The role of market structure for income tax rates. The figure plots market correction term $-E(n)$, defined in Proposition 2, against corresponding labor earnings $z(n)$

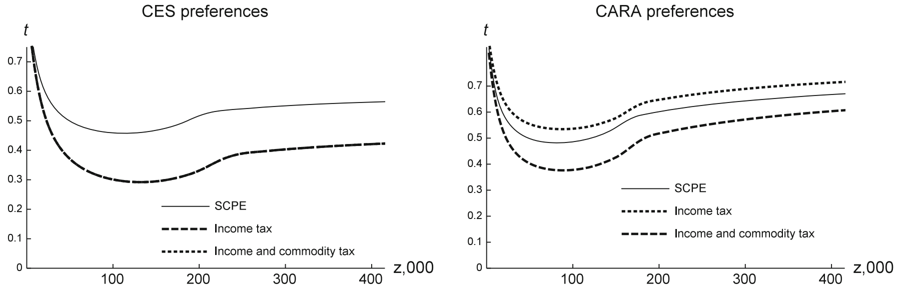

**Fig. 3** Income tax rates for CES and CARA preferences. The income tax rate schedules for the policy with income and commodity tax (dotted line) and income tax (dashed line) are found from Propositions 1 and 2, respectively. The SCPE tax schedule is found from the solution to problem (20)

As the social markup is different from the price markup (Proposition 1), income taxation needs to be complemented with commodity or firm taxation to achieve the social optimum. Comparing the policy outcomes obtained under Expo-Power and CARA preferences, we observe larger commodity subsidies under CARA preferences, 0.016 vs 0.012 (see Table 1), which imply a larger market inefficiency related to the variety effect. As a result, and unlike with Expo-Power preferences, we obtain increases in income tax rates above the SCPE benchmark. For the policy regime with only income taxes we observe a larger market correction term $-E(n)$ under CARA preferences (see the dashed line in Fig. 2) and, accordingly, smaller reductions in tax rates compared to the SCPE benchmark. Further analysis of CARA preferences provided in Table 1 reveals that the qualitative difference in economic outcomes between the two policy regimes is similar to that obtained under Expo-Power preferences.

Lastly, in Fig. 4 we compare income tax schedules among the preference types studied for a given policy regime. First, consumer preferences matter for policy design directly through workers' behavioral response, which is captured by the SCPE benchmark with an exogenous market structure (left panel). We observe that relative to the Expo-Power preferences the income tax schedule becomes more progressive for CARA preferences and less progressive for CES preferences. Second, with an endogenous market structure preferences can also matter indirectly through their influence

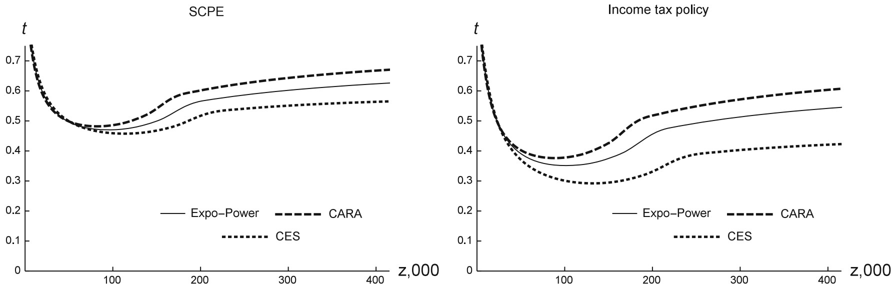

**Fig. 4** Income tax rates and preferences. For each type of preference considered, the tax schedule for the SCPE benchmark (left panel) is found from the solution to problem (20) and for Income tax policy from Proposition 2

on the market outcome. In the right panel of Fig. 4, we plot the tax schedules under the policy regime with income taxation. We observe that compared to the SCPE benchmark the differences in tax schedules are qualitatively similar though substantially amplified. In other words, when the market structure is endogenous preferences play a more profound role for income tax policy than in the case of a fixed market structure.

## 5 Conclusion

In this paper, we study the problem of tax policy design when accounting for the price and variety effects on workers' welfare arising from the entry and output inefficiencies of monopolistic market structure. Our particular focus is on the implications of market corrections for workers' income taxation. We demonstrate that due to inefficient market entry income taxation needs to be complemented with market intervention, via commodity or firm taxes, to achieve the constrained social optimum. Consequently, income taxes need to be adjusted to account for not only noncompetitive markups but also for the budgetary consequences of market intervention. Numerical simulations demonstrate that in the case of market corrections with commodity taxation the net effect of market intervention on income taxes is nearly zero, apart from some regressive changes in the tax structure. In particular, while the price effect of noncompetitive markups exerts a downward pressure on income taxes to stimulate labor supply and more production, additional tax revenue needed for efficient market entry can reverse this pressure. Overall, we estimate that the failure to account for the price and variety effects results in a welfare loss of 1.77%. Following practical examples, we also study a policy regime that is solely based on income taxation. For such regime, the conflicting goals of correcting for the price and variety effects imply departures from the constrained social optimum, which we estimate at 0.55%. However, this policy regime results in lower and less regressive income taxes than those obtained under the regime with income and commodity taxation and also in a substantially smaller government. Lastly, we examine the role of consumer preferences for policy outcomes and show that it is amplified by an endogenous market structure and, as a result, so are inaccuracies resulting from misspecified preferences.

**Acknowledgements** We are grateful to the referees and conference participants at the Third International Workshop "Market Studies and Spatial Economics" (Moscow) for useful comments and suggestions.

**Open Access** This article is licensed under a Creative Commons Attribution 4.0 International License, which permits use, sharing, adaptation, distribution and reproduction in any medium or format, as long as you give appropriate credit to the original author(s) and the source, provide a link to the Creative Commons licence, and indicate if changes were made. The images or other third party material in this article are included in the article's Creative Commons licence, unless indicated otherwise in a credit line to the material. If material is not included in the article's Creative Commons licence and your intended use is not permitted by statutory regulation or exceeds the permitted use, you will need to obtain permission directly from the copyright holder. To view a copy of this licence, visit http://creativecommons.org/licenses/by/4.0/.

## Appendix

In this Appendix, we provide proofs for the propositions stated in the main body of the paper.

### Proof of Proposition 1

First, we show that optimal tax policy leads to the constrained social optimum where workers' productivity is their private information but firm ownership is public. We define the constrained social optimum $(q(n), \ell(n), N)$ as the solution to the welfare maximization problem

$$\max_{q(n),\ell(n),N} \int [Nu(q(n)) - c(\ell(n))]\psi(n)dF(n)$$

subject to the incentive compatibility constraint

$$U(n) = Nu(q(n)) - c(\ell(n)) = \max_{n'} Nu(q(n')) - c(n'\ell(n')/n)$$

and the resource constraint

$$\int [n\ell(n) - kNq(n)]dF(n) - NK \geq G.$$

Using the envelope theorem, we can rewrite the incentive compatibility condition as

$$U'(n) = \frac{\ell(n)}{n}c'(\ell(n)).$$

Hence, the public authority's problem becomes

$$\max_{U(n),\ell(n),N} \int \psi(n)U(n)dF(n)$$

subject to

$$U'(n) - \frac{\ell(n)}{n} c'(\ell(n)) = 0$$
$$\int [n\ell(n) - kNr(U(n), \ell(n), N)] dF(n) - NK = G.$$

Using $\mu(n)$ and $\lambda$ as the respective Lagrange multipliers of the two constraints, we obtain that the constrained social optimum is characterized by the following first-order conditions

$$U(n) : \left(\psi(n) - \frac{\lambda k}{u'(q(n))}\right) f(n) = \mu'(n) \tag{24}$$

$$\ell(n) : \lambda \left(n - k\frac{c'(\ell(n))}{u'(q(n))}\right) f(n) = \mu(n)(c' + \ell(n)c'')/n \tag{25}$$

$$N : \int \frac{u(q(n))}{u'(q(n))/k} f(n)dn - (kQ + K) = 0. \tag{26}$$

Now consider the public authority's problem stated in (6) with private firm ownership. Using $\alpha$ and $\beta$ as the Lagrange multipliers for constraints (9) and (10) and denoting

$$Z(n) = \lambda N(p - s) - \alpha(p - k - s) - \beta \partial\eta/\partial q(n),$$

we can write the first-order conditions for the optimal income and firm taxation as

$$U(n) : \left(\psi(n) - \frac{Z(n)}{Nu'(q(n))}\right) f(n) - \mu'(n) = 0 \tag{27}$$

$$\ell(n) : \left(\lambda n - \frac{Z(n)c'(\ell(n))}{Nu'(q(n))}\right) f(n) - \mu(n)(c' + \ell c'')/n = 0 \tag{28}$$

$$p : \int \left(-\lambda Nq(n) + \alpha q(n) - \beta\frac{k + s}{p^2}\right) f(n)dn = 0 \tag{29}$$

$$N : \int \left(-\lambda(p - s)q(n) + \lambda S - Z(n)\frac{-u(q(n))}{Nu'(q(n))}\right) f(n)dn = 0 \tag{30}$$

$$s : \int \left(\lambda Nq(n) - \alpha q(n) + \frac{\beta}{p}\right) f(n)dn = 0 \tag{31}$$

$$S : \lambda N - \alpha \ = 0. \tag{32}$$

We now consider two scenarios: (1) the optimal income taxation with the optimal entry subsidy, but without commodity taxation: $s = 0$; and (2) the optimal income taxation with the optimal commodity taxation, but without entry subsidy: $S = 0$. For the first scenario, Eq. (31) is not present, whereas Eqs. (32) and (29) then imply $\alpha = \lambda N$ and $\beta = 0$ and, in turn, $Z(n) = \lambda Nk$. Taking this into account, we obtain

$$U(n) : \left(\psi(n) - \frac{\lambda k}{u'(q(n))}\right) f(n) - \mu'(n) = 0 \tag{33}$$

$$\ell(n) : \lambda \left( n - \frac{kc'(\ell(n))}{u'(q(n))} \right) f(n) - \mu(n)(c' + \ell c'')/n = 0 \quad (34)$$

$$N : \int \left( -(K + kq(n)) + \frac{ku(q(n))}{u'(q(n))} \right) f(n) dn = 0, \quad (35)$$

where the last equation follows from (30) and the zero profit condition $(p - k)Q - K - S = 0$. Hence, workers' utility, labor supply, and firms' production coincide with the ones in the constrained social optimum characterized by (24)–(26).

In the second scenario with $S = 0$, Eq. (32) is not present, whereas adding (31) and (29) yields

$$\beta \frac{p - k - s}{p^2} = 0.$$

The latter implies that $\beta = 0$ ($p = k + s$ would violate the free entry condition) and, therefore, $\alpha = \lambda N$ and, in turn, $Z(n) = \lambda N k$. Hence, the public authority can achieve worker's utility level, labor supply, and firms' production as in the constrained social optimum. This proves part (i) of the proposition.

The optimal income tax rates are found from Eqs. (27) and (28) and as these equations are the same in each scenario considered, the presence of commodity or entry taxation has no impact on optimal tax rates. Recall that we have from the individual utility maximization condition in (2)

$$1 - T'(n\ell(n)) = \frac{pc'(\ell(n))}{nu'(q(n))}$$

or, using $t(n\ell(n)) = T'(n\ell(n))$,

$$\frac{t(n\ell(n))}{1 - t(n\ell(n))} = \frac{nu'(q(n))}{pc'(\ell(n))} - 1.$$

From (34), we can derive that

$$\frac{nu'(q(n))}{c'(\ell(n))} = \frac{u'(q(n))\mu(n)(1 + \ell c''(\ell(n))/c'(\ell(n)))}{\lambda n f(n)} + k.$$

we obtain

$$\frac{t(n\ell(n))}{1 - t(n\ell(n))} = \frac{u'(q(n))\mu(n)(1 + \ell c''(\ell(n))/c'(\ell(n)))}{\lambda p n f(n)} - \frac{p - k}{p}.$$

The integration of (33) from $n$ to $\overline{n}$ and the transversality condition $\mu(\overline{n}) = 0$ yield

$$\mu(n) = \int_n^{\overline{n}} \left( \frac{\lambda k}{u'(q(n'))} - \psi(n') \right) f(n') dn'.$$

Using the marginal utility of income $\kappa(n) = u'(q(n))/p$, we derive

$$\mu(n) = \int_n^{\overline{n}} \left( \frac{\lambda k}{\kappa(n')p} - \psi(n') \right) f(n')dn'.$$

To derive labor supply elasticities $\zeta^u$ and $\zeta^c$, we follow Saez (2001) by linearizing the individual budget constraint as $pNq = w\ell + R$, where $w = n(1-t)$ is a net wage and $R$ is virtual (non-labor) income. Then, the first-order condition (2) implicitly determines labor supply $\ell = \ell(w, R)$ for a given market structure. The uncompensated labor supply elasticity is determined by $\zeta^u = (d\ell/dw)(w/\ell)$ and the compensated labor supply elasticity is found from the Slutsky equation $\zeta^c = \zeta^u - \tau$, where $\tau = wd\ell/dR$ is the income effect. We obtain the uncompensated and compensated labor supply elasticities equal to

$$\zeta^u = -\frac{(u''/N)(c'/u')^2 + c'/\ell}{(u''/N)(c'/u')^2 - c''} \qquad \zeta^c = -\frac{c'/\ell}{(u''/N)(c'/u')^2 - c''}$$

yielding $1 + \ell(n)c''/c' = (1+\zeta^u)/\zeta^c$. Hence, we have

$$\frac{t(n\ell(n))}{1 - t(n\ell(n))} = \frac{1+\zeta^u}{\zeta^c} \frac{\kappa(n) \int_n^{\overline{n}} \left( \frac{\lambda k}{\kappa(n')p} - \psi(n') \right) f(n')dn'}{\lambda n f(n)} - \frac{p-k}{p},$$

which is equivalent to the formula from the proposition.

Regarding the sign of the optimal entry tax $S$, from the zero profit condition we have

$$S = pQ - kQ - K$$

or, using Eq. (35) and the definition of social markup $\delta$ in (11),

$$\begin{aligned} S &= pQ - k \int \frac{u(q(n))}{u'(q(n))} f(n)dn \\ &= Q\left(p - \frac{k}{1-\delta}\right). \end{aligned}$$

Thus, we obtain $S \lesseqqgtr 0$ if $\frac{p-k}{p} \lesseqqgtr \delta$ as required. Regarding the sign of the optimal commodity tax $s$, the zero profit condition and Eq. (35) imply

$$sQ = Q\left(p - \frac{k}{1-\delta}\right),$$

from which we find

$$\delta = \frac{p-s-k}{p-s} = \eta \frac{p}{p-s},$$

where the last equality is obtained from the optimal price condition in program (6). Hence, we obtain $s \lesseqqgtr 0$ if $\delta \lesseqqgtr \eta$.

## Proof of Proposition 2

As before, we define

$$Z(n) = \lambda Np - \alpha(p-k) - \beta\partial\eta/\partial q(n).$$

Then, the first-order conditions for the public authority's maximization problem are given by

$$U(n): \left(\psi(n) - \frac{Z(n)}{Nu'(q(n))}\right) f(n) = \mu'(n) \tag{36}$$

$$\ell(n): \left(\lambda n - \frac{c'Z(n)}{Nu'(q(n))}\right) f(n) = \frac{\mu(n)(c' + \ell(n)c'')}{n} \tag{37}$$

$$p: \int \left(-\lambda Nq(n) + \alpha q(n) - \frac{k\beta}{p^2}\right) dF(n) = 0 \tag{38}$$

$$N: \int \left(-\lambda pq(n) + \frac{Z(n)u(q(n))}{Nu'(q(n))}\right) dF(n) = 0. \tag{39}$$

From (37), we have

$$\lambda\left(\frac{nu'(q(n))}{pc'(\ell(n))} - 1\right) + \frac{\alpha(p-k) + \beta\partial\eta/\partial q(n)}{pN} = \frac{u'(q(n))}{p}\frac{\mu(n)(1 + \ell(n)c''/c')}{f(n)n}.$$

Taking into account that

$$\frac{t(n\ell(n))}{1 - t(n\ell(n))} = \frac{nu'(q(n))}{pc'(\ell(n))} - 1,$$

we have

$$\frac{t(n\ell(n))}{1 - t(n\ell(n))} = \frac{u'(q(n))\mu(n)(1 + \ell(n)c''/c')}{\lambda pnf(n)} - \frac{\alpha(p-k) + \beta\partial\eta/\partial q(n)}{\lambda pN}.$$

From (36), we obtain that

$$\mu(n) = \int_n^{\bar{n}} \left(\lambda p\frac{1 - E(n')}{u'(q(n'))} - \psi(n')\right) f(n')dn',$$

where

$$E(n) = \frac{\alpha(p-k) + \beta\partial\eta/\partial q(n)}{\lambda pN}.$$

Hence, the marginal tax is given by

$$\frac{t(n\ell(n))}{1-t(n\ell(n))} = \frac{1+\zeta^u}{\zeta^c}\frac{\kappa(n)}{nf(n)}\int_n^{\bar{n}} \frac{1-\kappa(n')\psi(n')/\lambda - E(n')}{\kappa(n')} f(n')dn' - E(n).$$

Regarding the sign of multiplier $\beta$, from (38) we have

$$Q(-\lambda N + \alpha) = \frac{\beta k}{p^2}, \tag{40}$$

where $Q$ is the aggregate demand for a variety. This implies $sign(\beta) = sign(\alpha - \lambda N)$.
